# Antibiotic therapy in patients with amniotic fluid sludge and risk of preterm birth: a meta-analysis

**DOI:** 10.1007/s00404-023-07045-1

**Published:** 2023-04-25

**Authors:** I. Sapantzoglou, V. Pergialiotis, I. Prokopakis, A. Douligeris, S. Stavros, P. Panagopoulos, M. Theodora, P. Antsaklis, G. Daskalakis

**Affiliations:** 1 1st Department of Obstetrics and Gynecology, ‘Alexandra’ Hospital, National and Kapodistrian University of Athens, 2-4, Lourou Str, 11528 Athens, Greece; 2 3rd Department of Obstetrics and Gynecology, Attikon University Hospital, National and Kapodistrian University of Athens, Athens, Greece

**Keywords:** Amniotic fluid, Antibiotics, Preterm delivery, Preterm labor, Sludge

## Abstract

**Purpose:**

Amniotic Fluid Sludge (AFS) has been theorized to be sonographic evidence of an underlying infection/inflammation and studies have concluded that approximately 10% of the patients who show signs of preterm labor with intact membranes have an underlying intraamniotic infection, mostly subclinical, carrying an increased risk for preterm birth with its subsequent neonatal and maternal complications. The purpose of the present systematic review is to evaluate the impact of antibiotic therapy on preterm birth rates of women diagnosed with AFS.

**Methods:**

We searched Medline, Scopus, the Cochrane Central Register of Controlled Trials CENTRAL, Google Scholar, and Clinicaltrials.gov databases for relevant articles published until the 30th of September 2022. Observational studies (prospective and retrospective) that evaluated the impact of antibiotics on preterm delivery rates of patients with AFS were considered eligible for inclusion. Statistical meta-analysis was performed with RStudio and we calculated pooled risk ratios (OR) and 95% confidence intervals (CI). To evaluate the information size, we performed trial sequential analysis (TSA) and the methodological quality of the included studies was assessed using RoBINS tools.

**Results:**

Overall, four retrospective cohort studies were included in the present systematic review and 369 women were enrolled. We demonstrated that preterm delivery prior to 34, 32 and 28 weeks of gestational age was comparable among the groups of women that had antibiotics and those that did not (OR: 0.34, 95% CI 0.05, 2.14, 0.40 [0.09, 1.66], 0.35 [0.08, 1.58], respectively) but the statistical heterogenicity of the studies included was high for every gestational period that was examined.

**Conclusions:**

According to our study, we cannot conclude that the use of antibiotics in women with amniotic fluid sludge benefit the prognostic risk to deliver prematurely. It is quite clear that data from larger sample sizes and more well adjusted and designed studies are needed.

## What does this study add to the clinical work

**Table:** 

Our study accumulates the available data and evaluates the impact of antibiotic therapy on preterm birth rates of women diagnosed with AFS. The potential benefit of antibiotics in AFS patients should be reevaluated and more data need to be collected for the results to be concrete and for antibiotics to be justified in a universal manner.	

## Introduction

Preterm birth is the leading cause of neonatal morbidity and mortality affecting approximately 10–12% of all pregnancies [1]. It accounts for more than 70% of all neonatal deaths and its etiology remains multifactorial [2, 3]. Nevertheless, modern obstetrical research has made serious improvements in identifying the patients at risk, associated risk factors and implementing clinical interventions to reduce the risk of prematurity. Several factors have been implicated in preterm birth and one such factor is the sonographic finding of amniotic fluid “sludge’’ (AFS) [4].

Amniotic fluid sludge is defined as the sonographic finding of hyperechogenic, free floating matter in close proximity to the uterine cervix [5]. Current evidence suggests that the incidence of sludge is 1% and may increase up to 23% in population at high risk for preterm delivery [5, 6]. A growing body of literature addresses AFS as a biomarker for intra-amniotic infection/inflammation and considers it an independent risk factor for preterm prelabor rupture of membranes (PPROM), as well as spontaneous preterm delivery [7]. According to Romero et al., microorganisms can invade the membranes and the fetus either in an ascending way through the lower genital tract or via hematogenous spread from maternal bacteremia [8]. The most common isolated organisms are Ureoplasma urealyticum, Fusobacterium species and Mycoplasma Hominis [8]. The subsequent development of inflammatory response which triggers the accumulation of cytokines, such as IL-1,IL-6,TNF-a, chemokines and metalloproteinases in the membranes, the umbilical cord and the placenta has been implicated in the stimulation of contractions, cervical ripening and rupture of membranes [8–10].

In our previous systematic review, we noted that the predominant morbidity that is associated with AFS is preterm birth (PMID: 32,367,525). Specifically, the majority of the evidence suggests that the presence of sludge increases the risk of preterm birth < 37, < 34, < 32 and < 28 weeks respectively [11]. The impact of antibiotics on the pregnancy course of women with amniotic fluid sludge became a matter of investigation over the last years. Initially, case reports indicated that amniocentesis of these patients was indicative of infection [12, 13]. However, the actual type of bacteria remained a challenge to identify and a generalized approach of treatment such as in cases diagnosed with PPROM has not been adopted. Since then, clinical trials have been published, however, still the scientific community has not reached specific recommendations concerning the importance of antibiotic coverage of these cases [14–17].

In the present metanalysis we accumulate for the first time in the literature the available evidence to help clarify the impact of antibiotic therapy on preterm birth rates of women diagnosed with AFS.

### Objective

The purpose of the present systematic review is to summarise the available published data on the impact of antibiotic therapy on preterm birth rates of women diagnosed with AFS.

### Methods

The present meta-analysis was designed according to the Preferred Reporting Items for Systematic Reviews and Meta-Analyses (PRISMA) guidelines. The study was based in aggregated data that have been already published in the international literature. Patient consent and institutional review board approval were not retrieved as they are not required in this type of studies. The study`s protocol was published in PROSPERO (International prospective register of systematic reviews) prior to the conduct of this meta analysis (Registration number: CRD CRD42022337730).

### Eligibility criteria, information sources, search strategy

The eligibility criteria for the inclusion of studies were predetermined. Observational studies (prospective and retrospective) as well as randomized trials that evaluated the impact of antibiotics (irrespective of the regimen used) on preterm delivery rates of patients with AFS were considered eligible for inclusion. The type of administered antibiotics was anticipated to vary among studies included as indicated in the discussion section of the present article; hence, we opted to include all studies and in the presence of sufficient evidence provide a subgroup-analysis to help indicate the optimal combination. Case reports, experimental studies and conference proceedings were excluded from the present meta-analysis.

**Fig. 1:**
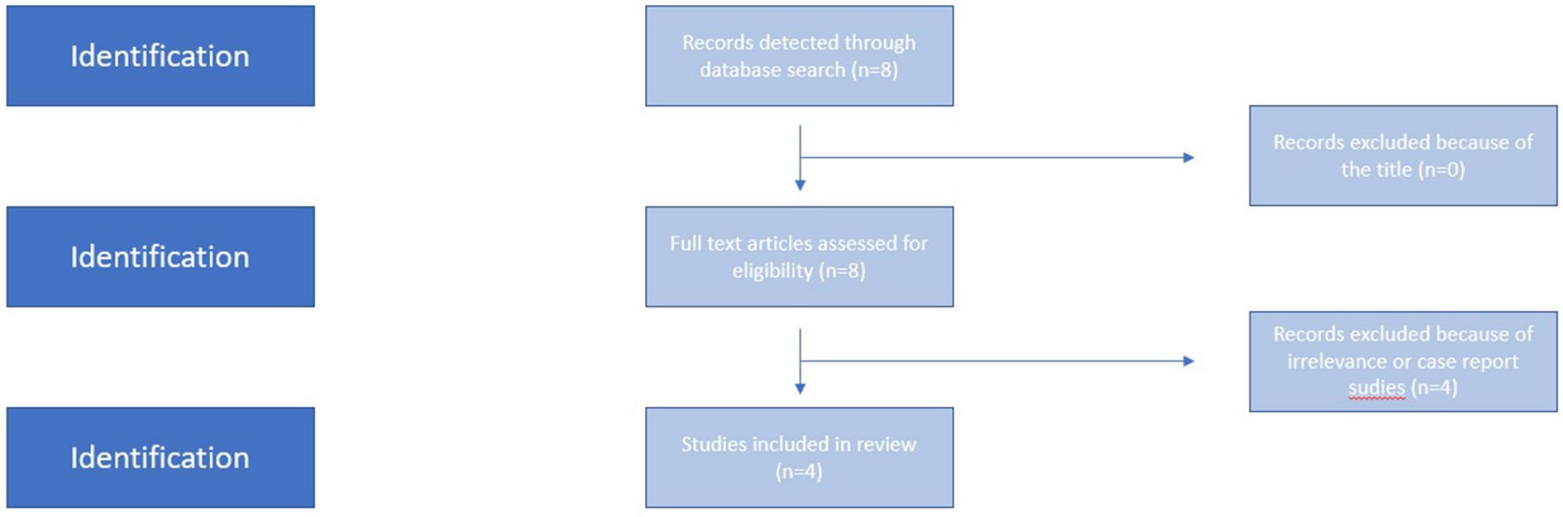
Search strategy

We used the Medline (1966–2021), Scopus (2004–2021), Clinicaltrials.gov (2008–2021), EMBASE (1980–2021), Cochrane Central Register of Controlled Trials CENTRAL (1999–2021) and Google Scholar (2004–2021) databases in our primary search along with the reference lists of electronically retrieved full-text papers. The date of our last search was set at September 30, 2022. Our search strategy included the text words “amniotic fluid; sludge; antibiotic;preterm delivery;preterm labor” and is briefly presented in Fig. 1. The search identified 8 potentially relevant studies, but 4 were excluded because they were non-relevant articles or case reports and, in total, only 4 peer-reviewed papers were considered for inclusion in the current metaanalysis [14–17].

### Study selection

Studies were selected in three consecutive stages. Following deduplication, the titles and abstracts of all electronic articles were screened by two authors (I.S. and V.P.) to assess their eligibility. The decision for inclusion of studies in the present meta-analysis was taken after retrieving and reviewing the full version of articles that were considered potentially eligible. All studies that evaluated the impact of antibiotic therapy among patients diagnosed with amniotic sludge and reported preterm birth rates were considered eligible for inclusion. Discrepancies in the prescribed antibiotic therapy (type of antibiotic and duration) were anticipated; hence, predetermination of a specific regimen was not considered. Discrepancies that arose concerning the eligibility of retrieved studies were resolved by consensus from all authors.

### Data extraction

Outcome measures were predefined during the design of the present systematic review. Data extraction was performed using a modified data form that was based in Cochrane`s data collection form for intervention reviews for RCTs and non-RCTs. We predetermined as primary outcome differences in the odds of preterm birth < 34 weeks of gestation. The odds of preterm delivery < 32, < 28 weeks and < 37 weeks were evaluated as secondary outcomes as well as the odds of developing chorioamnionitis. An additional subanalysis was performed to focus on the high risk pregnant women based on history of preterm birth, the presence of uterine contractions, short cervix, Mullerian wolff malformations, late miscarriages and cervical conization whenever those data were provided.

### Data synthesis

Statistical meta-analysis was performed with RStudio using the *meta* function (RStudio Team (2015). RStudio: Integrated Development for R. RStudio, Inc., Boston, MA URL http://www.rstudio.com/). Statistical heterogeneity was not considered during the evaluation of the appropriate model (fixed effects or random effects) of statistical analysis as the considerable methodological heterogeneity (Table 1) did not leave space for assumption of comparable effect sizes among studies included in the meta-analysis [4]. Confidence intervals were set at 95%. We calculated pooled risk ratios (OR) and 95% confidence intervals (CI) with the Hartung-Knapp-Sidik-Jonkman instead of the traditional Dersimonian-Laird random effects model analysis (REM). The decision to proceed with this type of analysis was taken after taking into consideration recent reports that support its superiority compared to the Dersimonian-Laird model when comparing studies of varying sample sizes and between-study heterogeneity. Publication bias was not assessed due to the small number of included studie.

**Table 1:** Summary of studies included

Author	Year	Study Design	Inclusion criteria	Exclusion criteria	Antibiotic Regimen	Primary outcomes
Jin et al.	2021	Retrospective Cohort	Singleton Pregnancy, 15–32 weeks of gestation, presence of uterine contractions, intact membranes, presence of AFS on ultrasound	Refusal to participate, impending preterm delivery, multiple gestations, PPROM, fetal anomalies, placenta previa, iatrogenic preterm delivery for maternal/fetal reasons	IV ceftriaxone 1 g/24 h, PO clarithromycin (500 mg)/12 h, IV metronidazole (500 mg)/8 h for maximum 4 weeks	Eradication of AFS, Preterm Delivery < 28 weeks, Preterm Delivery < 32 weeks, Preterm Delivery < 34 weeks
Cuff et al.	2019	Retrospective Cohort	Singleton Pregnancy, 15–25 weeks of gestation, intact membranes, presence of AFS on ultrasound	Refusal to participate, impending preterm delivery, multiple gestations, PPROM prior to the assessment, fetal anomalies, placenta previa or suspicion of accreta, active bleeding, incomplete data	PO azithromycin (500 mg)/24 h on D1 followed by PO azithromycin (250 mg)/24 h on D2-D5 or PO Moxifloxacin (400 mg)/24 h for 5 days	Preterm delivery < 37 weeks gestational age
Hatanaka et al.	2019	Historically controlled observational study	Singleton Pregnancy, 16–26 weeks of gestation, intact membranes, presence of AFS on ultrasound	Congenital fetal malformation, placenta previa, preterm labor, vaginal bleeding or a history of cervical previa, preterm labor, vaginal bleeding or a history of cervical cerclage	Low risk pregnant women: PO clindamycin (300 mg)/6 h and cephalexin(500 mg)/6 h for 7 days, High risk pregnant women: IV clindamycin (600 mg)/8 h and cephazolin(1gr)/8 h followed by 5 days of PO treatment	Preterm Delivery < 28 weeks, Preterm Delivery < 32 weeks, Preterm Delivery < 34 weeks, Preterm delivery < 37 weeks gestational age at both high and low risk women
Fuchs et al.	2014	Retrospective case–control and Historically controlled observational study	Singleton Pregnancy, 15–32 weeks of gestation, threatened preterm labour, asymptomatic women, asymptomatic women at high risk (history of spontaneous preterm delivery or previous mid-trimester loss), intact membranes, presence of AFS on ultrasound	Refusal to participate, multiple gestations, PPROM prior to the assessment, fetal anomalies, placenta previa, iatrogenic preterm delivery for maternal/fetal reasons	PO azithromycin(500 mg)/24 h on D1 followed by PO azithromycin (250 mg)/24 h on D2-D5	Preterm Delivery < 28 weeks, Preterm Delivery < 32 weeks, Preterm Delivery < 34 weeks

*AFS* Amniotic Fluid Sludge, *PPROM* Preterm Premature Rupture of Membranes, *PO* Per OS

The potential presence of small-study effects was planned to be evaluated with Rücker’s Limit Meta-Analysis and the possibility of p-hacking with inspection of the results of the p-curve analysis. None of these analyses as the number of studies did not suffice to provide robust results.

Prediction intervals (PI) were calculated using the *meta* function in RStudio, to evaluate the estimated effect that is expected to be seen by future studies in the field. The estimation of prediction intervals considers the inter-study variation of the results and express the existing heterogeneity at the same scale as the examined outcome.

To evaluate the information size, we performed trial sequential analysis (TSA) which permits investigation of the type I error in the aggregated result of meta-analyses performed for primary outcomes that were predefined in the present meta-analysis. A minimum of 3 studies was considered as appropriate to perform the analysis. Repeated significance testing increases the risk of type I error in meta-analyses and TSA has the ability to re-adjust the desired significance level using the O` Brien-Flemming a-spending function. Therefore, during TSA sequential interim analyses are performed that permit investigation of the impact of each study in the overall findings of the meta-analysis. The risk for type I errors was set at 5% and for type II errors at 20%. The TSA analysis was performed using the TSA v. 0.9.5.10 Beta software (http://www.ctu.dk/tsa/).

### Assessment of risk of bias

**Fig. 2:**
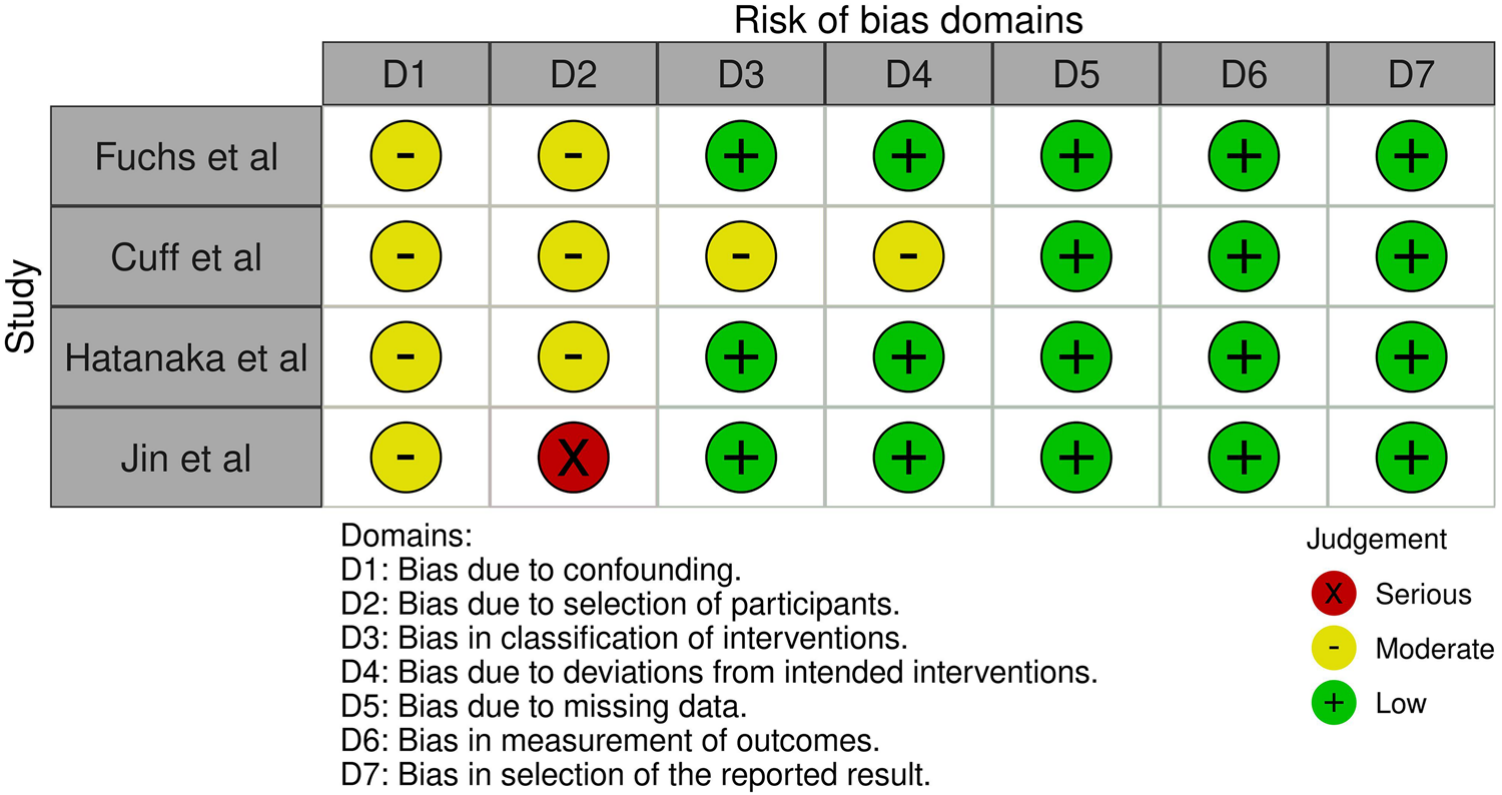
ROBINS-I tool for assessment of risk of bias

The quality of non-randomized trials was assessed with Risk of Bias in non-Randomized Trials (ROBINS-I) tool which incorporates 5 domains that investigated bias that arises (i) from confounders, (ii) from selection of participants, (iii) from selective reporting in intervention measures, (iv) from deviations from intended interventions, (v) due to missing data, (vi) from selective reporting in outcome measures and (vii) from selective reporting of outcomes (Fig. 2).

Quality of evidence was evaluated under the Grading of Recommendations Assessment, Development and Evaluation (GRADE) framework, ranging from very low to high. More specifically, credibility of evidence will be assessed by taking into account the following domains: study limitations, directness, consistency, precision and publication bias.

## Results

### Study selection and characteristics

Our search identified 8 potentially relevant studies, but 4 were excluded because they were non-relevant articles or case reports. Overall, 4 retrospective cohort studies were included in the present systematic review with 2 of them being historically controlled [14–17]. The search strategy is briefly presented in Fig. 1. The studies enrolled 369 women. Of those, 246 (66.6%) were administered antibiotic therapy and 123 (33.4%) controls were not. The methodological characteristics of included studies are summarized in Table 1 and the demographic characteristics of the patients analysed are depicted in Table 2. Similar timing of antenatal recording of AFS was observed among studies included (15–35 weeks). Significant variability was observed in the antibiotic schemes that were used among studies included. Documentation of the actual duration of used antibiotics was underreported. Factors that are known to predispose to preterm birth including cervical length, use of progesterone or other tocolytics were also underreported. All studies considered preterm birth rates as primary outcomes.

**Table 2:** Demographic characteristics documented on the studies included

		Cuff et al.		Fuchs et al.		Hatanaka et al.		Jin et al.	
		Treated (*n* = 51)	Non- treated (*n* = 46)	Treated (*n* = 63)	Non- treated (*n* = 14)	Treated (*n* = 22)	Non- treated (*n* = 64)	Treated (*n* = 30)	Non- treated (*n* = 28)
Ethnicity	African American	36 (70.6%)	31 (67.4%)	19 (35)	N/A	11 (50.5%)	32 (50.0%)	N/A	N/A
	Asian	N/A	N/A	N/A	N/A	1 (4.5%)	2 (3.1%)	N/A	N/A
	Caucasian	N/A	N/A	28 (52)	N/A	10 (45.5%)	30 (46.9%)	N/A	N/A
Maternal age		N/A	N/A	32	N/A	30.6	28.1	33.8	35.6
Nulliparous		13 (25.5%)	8 (17.4%)	13 (21%)	3 (21%)	8 (36.4%)	18 (28.1%)	14 (46.7%)	10 (35.7%)
History of PTB		24 (47.1%)	22 (47.8%)	36 (57)	4 (29)	N/A	N/A	8 (26.7%)	5 (17.9%)
17-OHPC administration		18 (36%)	15 (33%)	N/A	N/A	N/A	N/A	N/A	N/A
Vaginal progesterone administration		36 (71%)	29 (63%)	N/A	N/A	10 (45.5%)	41 (64.1%)	N/A	N/A
No progesterone supplementation		9 (18%)	11 (24%)	N/A	N/A	N/A	N/A	N/A	N/A
Mean BMI in 1st Trimester, kg/m2		33.7	32.8	26.7	N/A	26.5	28.4	25	25
Mean BMI at delivery, kg/m2		34.9	35.3	N/A	N/A	N/A	N/A	N/A	N/A
Tobacco use		4 (7.8%)	5 (10.9%)	N/A	N/A	7 (31.8%)	6 (9.4%)	N/A	N/A
Cerclage during index pregnancy		27 (52.9%)	20 (43.5%)	12 (19)	N/A	4 (18.2%)	4 (6.3%)	N/A	N/A
Mean GA cerclage placement, weeks		17.6	15.9	N/A	N/A	N/A	N/A	N/A	N/A
Sludge present at time of cerclage		14 (51.9%)	8 (40%)	N/A	N/A	N/A	N/A	N/A	N/A
Initial size of AFS (cm)		n/a	n/a	N/A	N/A	N/A	N/A	1.3	1.7
Mean GA at Dx of sludge, weeks		19.7	20	20	22	N/A	N/A	22.1	22.4
Mean TVCL at time of Dx of AFS, mm		18.9	22	25	19	N/A	N/A	13.5	10.8
Mean shortest TVCL recorded, mm		14.9	17	N/A	N/A	N/A	N/A	N/A	N/A
Duration of administration of antibiotics	n/a	N/A	N/A	N/A	N/A	N/A	21	21	15.6
Pessary after inclusion		N/A	N/A	N/A	N/A	1 (4.5%)	10 (15.6%)	N/A	N/A
Mullerian malformation		N/A	N/A	N/A	N/A	1 (4.5%)	3 (4.7%)	N/A	N/A
Cervical colonization		N/A	N/A	N/A	N/A	0 (0)	1 (1.6%)	N/A	N/A
First trimester vaginal bleeding		12 (23.5%)	10 (21.7%)	N/A	N/A	4 (18.2%)	14 (21.9%)	N/A	N/A
Average number of abortions		N/A	N/A	N/A	N/A	1.4	1.3	N/A	N/A

*PTB *Preterm Birth, *AFS*Amniotic Fluid Sludge, *BMI* Body Mass Index, *TVCL* Transvaginal Cervical Length, *17-OHPC* 17-Hydroxyprogesterone caproate

### Synthesis of results

**Fig. 3:**
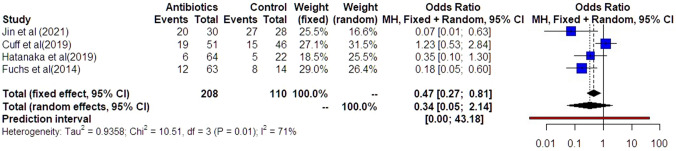
Forest plots of odds ratio for preterm delivery prior to 34 weeks in women with AFS that received antibiotics compared to women that did not receive with 95% confidence intervals (CI) and weighted pooled summary statistics using bivariate random-effects model

**Fig. 4:**
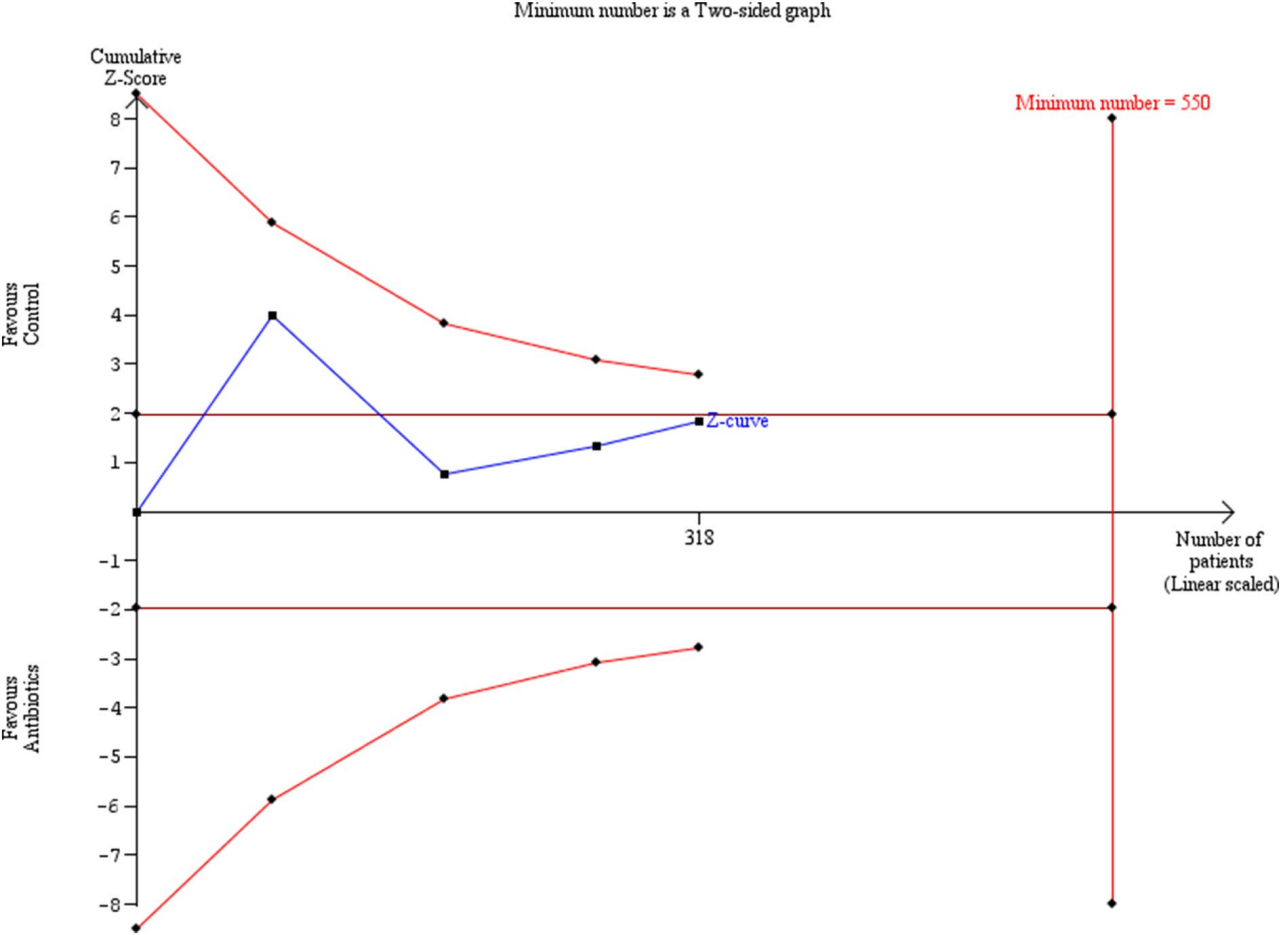
Trial sequential analysis of the indicated sample size needed to be obtained for adequate power for preterm delivery prior to 34 weeks

Preterm delivery prior to 34 weeks was comparable among the groups of women that had antibiotics and those that did not (OR: 0.34 [0.05, 2.14]) (Fig. 3). Statistical heterogenicity was high (*I*^2^ = 71%). Trial sequential analysis revealed that the required sample size (550 women) to reach safe conclusions was not reached (Fig. 4).

**Fig. 5:**
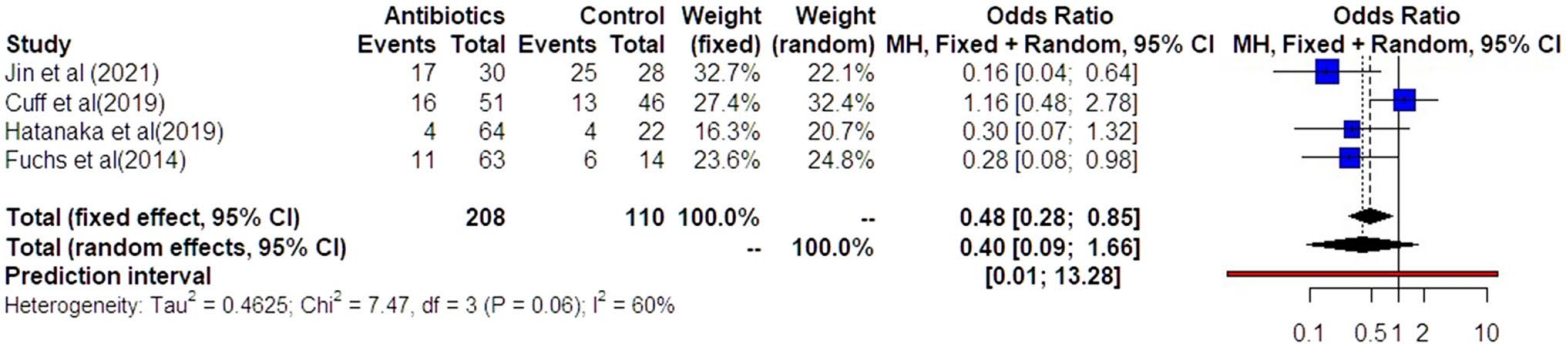
Forest plots of odds ratio for preterm delivery prior to 32 weeks in women with AFS that received antibiotics compared to women that did not receive with 95% confidence intervals (CI) and weighted pooled summary statistics using bivariate random-effects model

**Fig. 6:**
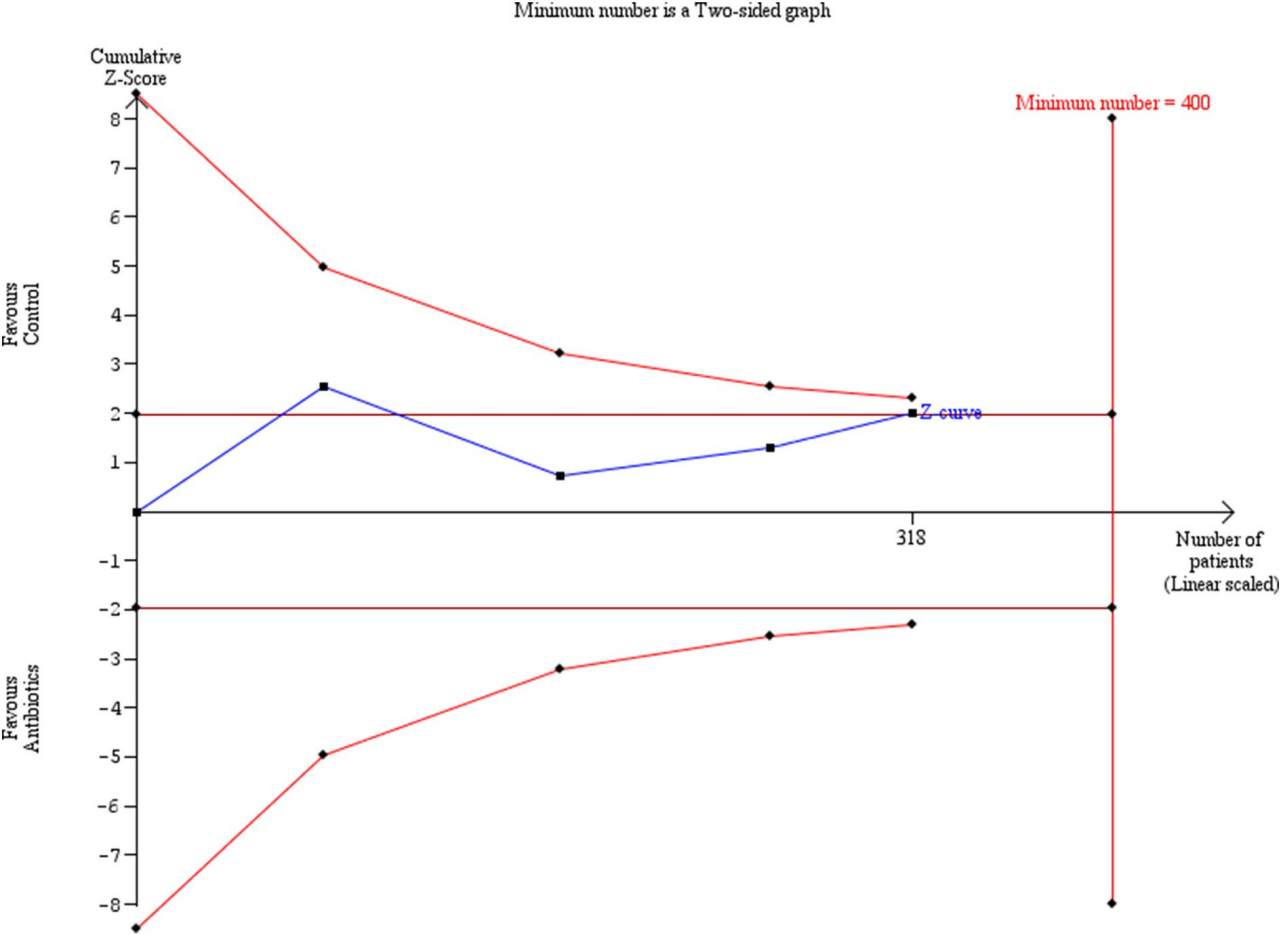
Trial sequential analysis of the indicated sample size needed to be obtained for adequate power for preterm delivery prior to 32 weeks

Preterm delivery prior to 32 weeks was also comparable among the groups of women that had antibiotics and those that did not (OR: 0.40 [0.09, 1.66]) (Fig. 5). Statistical heterogenicity was high (*I*^2^ = 60%) and the trial sequential analysis revealed that the required sample size (400 women) to reach concrete conclusions was not reached (Fig. 6).

**Fig. 7:**
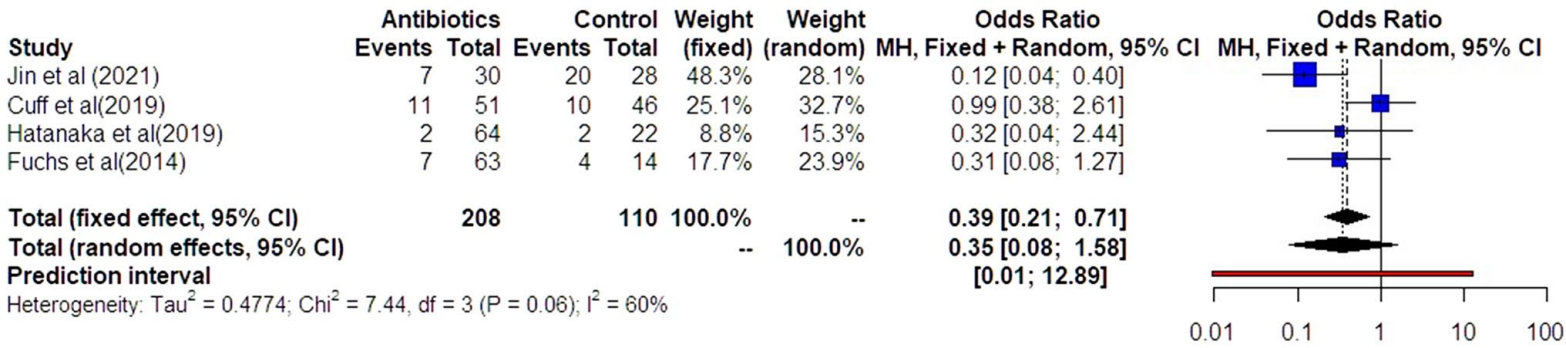
Forest plots of odds ratio for preterm delivery prior to 32 weeks in women with AFS that received antibiotics compared to women that did not receive with 95% confidence intervals (CI) and weighted pooled summary statistics using bivariate random-effects model

**Fig. 8:**
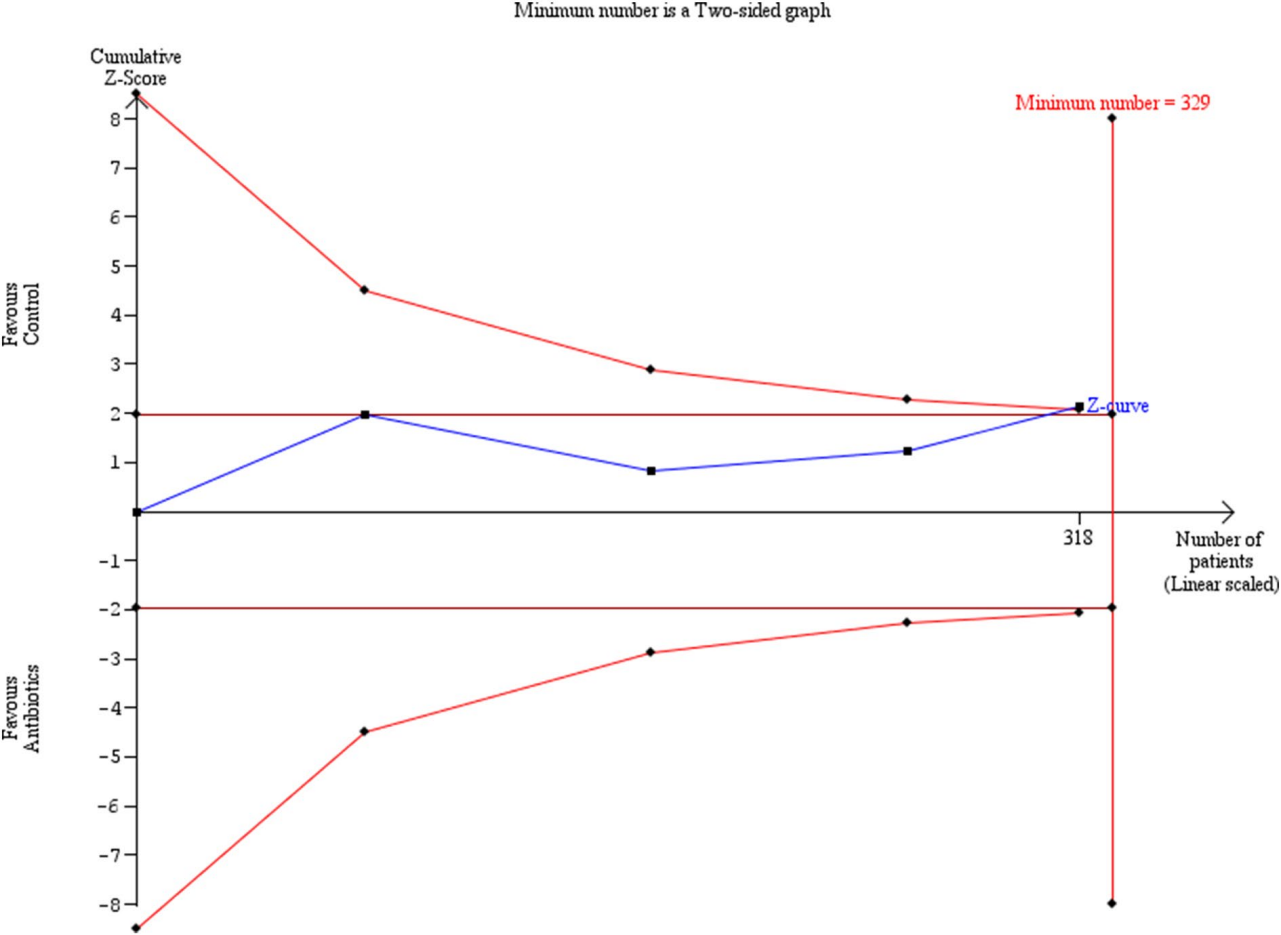
Trial sequential analysis of the indicated sample size needed to be obtained for adequate power for preterm delivery prior to 32 weeks

Similar results were observed in the investigation of preterm delivery prior to 28 weeks (Fig. 7). Preterm delivery did not show any statistically significant difference among the groups of women that had antibiotics and those that did not (OR: 0.35 [0.08, 1.58]). Statistical heterogenicity was high (*i*^2^ = 60%) and the trial sequential analysis revealed that the required sample size (329 women) to reach valid conclusions was, again, not reached (Fig. 8).

**Fig. 9:**
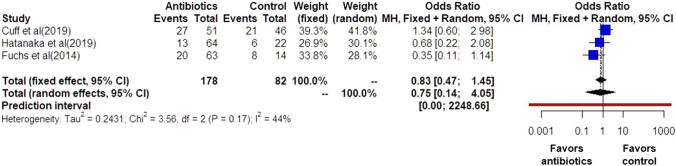
Forest plots of odds ratio for preterm delivery prior to 37 weeks in women with AFS that received antibiotics compared to women that did not receive with 95% confidence intervals (CI) and weighted pooled summary statistics using bivariate random-effects model

**Fig. 10:**
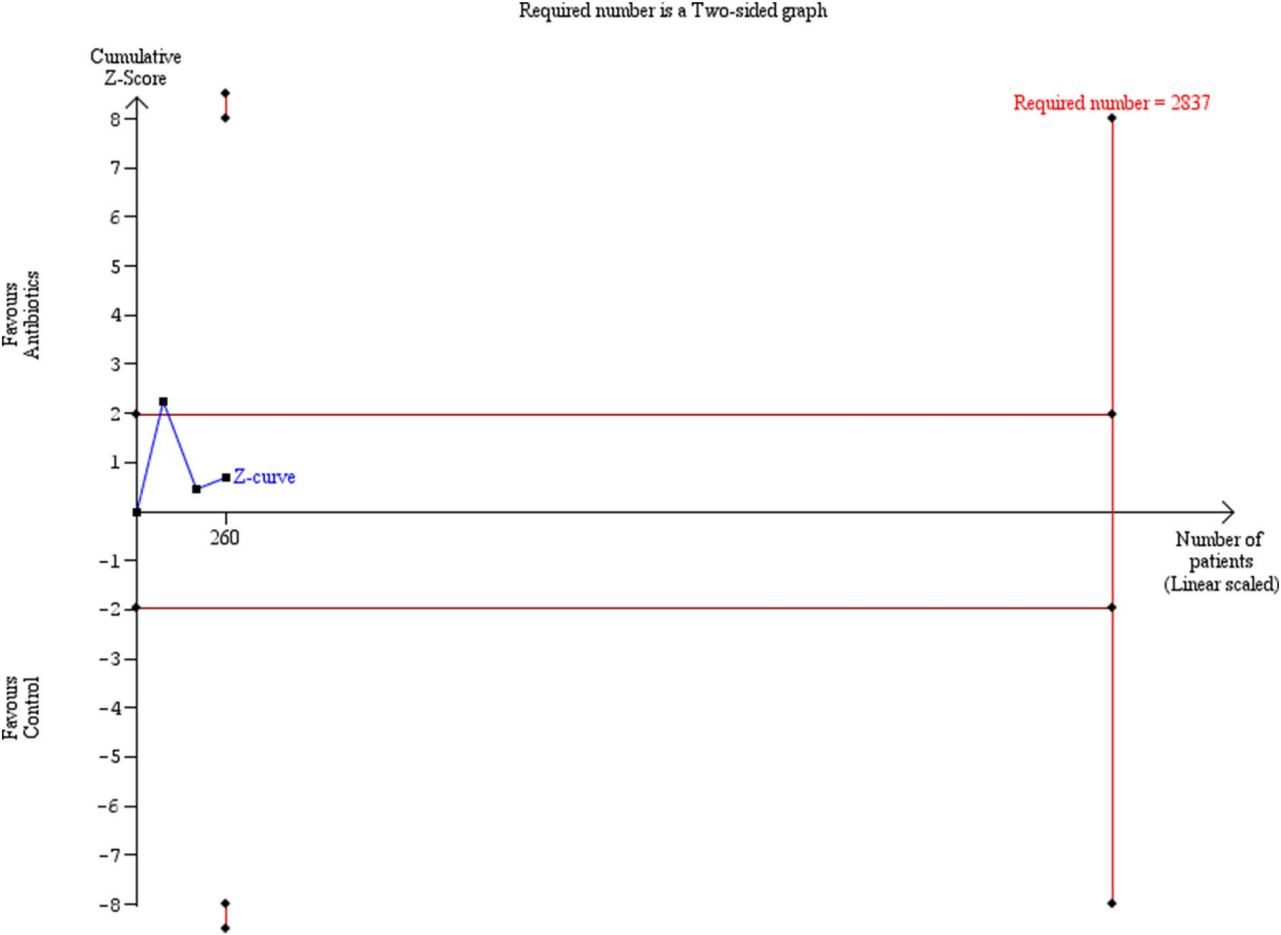
Trial sequential analysis of the indicated sample size needed to be obtained for adequate power for preterm delivery prior to 37 weeks

**Fig. 11:**
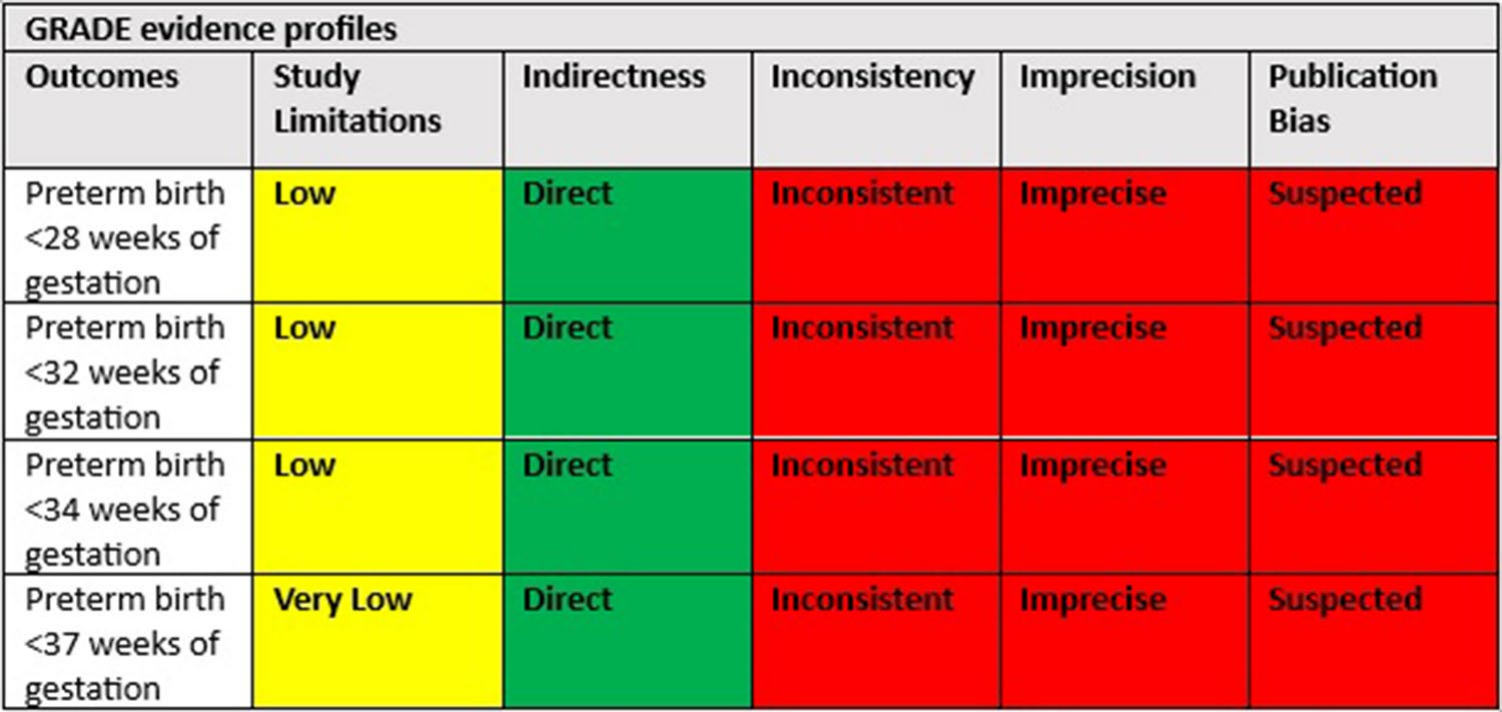
GRADE assessment of quality of evidence

Preterm delivery prior to 37 weeks was also comparable among the groups of women that had antibiotics and those that did not (OR: 0.75 [0.14, 4.05]) (Fig. 9). Statistical heterogenicity was high (*I*^2^ = 44%) and the trial sequential analysis revealed that the required sample size (2837 women) to reach concrete conclusions was not reached (Figs. 10, 11).

The additional subanalysis that was conducted to investigate the preterm birth rates among the high risk women that had antibiotics and those that did not revealed a statistical significant reduction of preterm birth < 28 weeks (OR: 0.20 [0.05, 0.83], *p*: 0.04, *I*^2^ = 0%) < 32 weeks (OR: 0.23 [0.10, 0.51] *p*: 0.02, *I*^2^ = 0%) and < 34 weeks (OR: 0.17 [0.05, 0.59] *p*: 0.02, *I*^2^ = 0%) and a non statistical significant improvement of preterm birth < 37 weeks (OR: 0.68 [0.11, 4.26] *p*: 0.45, *I*^2^ = 51%) (Table 3).

**Table 3:** Subanalysis of preterm delivery rates on high risk pregnant women and on the rates of chorioamnionitis

Table. Secondary outcomes			
Variable	Odds ratio (95% Confidence interval)	*p*-value	Heterogeneity (*I*-square)
Chorioamnionitis	0.57 (0.13, 2.48)	.24	0%
High risk < 28	0.20 (0.05, 0.83)	.04	0%
High risk < 32	0.23 (0.10, 0.51)	.02	0%
High risk < 34	0.17 (0.05, 0.59)	.02	0%
High risk < 37	0.68 (0.11, 4.26)	.45	51%

The subanalysis for the development of chorioamnionitis demonstrated a non significant improvement of chorioamnionitis among the women who received antibiotics and those that did not (OR:0.57 [0.13, 2.48] *p*:0.24, *I*^2^ = 0%) (Table 3).

## Comment

### Principal findings

The main objective of our study was to accumulate current evidence on the rates of preterm delivery in patients with sonographic evidence of amniotic fluid sludge (AFS) that received antibiotic treatment. Our metaanalysis was based on 4 primary studies which to our knowledge are the only retrospective cohort studies that have compared the rates of preterm delivery in patients with AFS that received antibiotics with those that did not. The findings of our study suggest that the use of antibiotics does not benefit the prognostic risk of delivering prematurely. The findings of our subanalysis on the preterm delivery rates of the high risk subgroups for prematurity suggested that antibiotics are associated with a statistical significant reduction for preterm labour < 28 weeks, < 32 weeks and < 34 weeks. An additional subanalysis on the raters of chorioamnionitis did not manage to demonstrate an improvement after the use of antibiotics. (Table 3).

### Strengths and limitations

To the best of ouf knowledge, this is the first meta analysis to address the impact of antibiotic therapy on preterm birth rates of women diagnosed with AFS. One of the main strengths of the current meta-analysis is the homogeneity of the nature of delivery. Hatanaka et al. included only spontaneous deliveries and Jinn et al. as well as, Fuchs et al. used iatrogenic preterm deliveries as an exclusion criterion. Cuff et al. do not state explicitly the nature of delivery but we believe it is safe to assume that,due to its study design and the preceding literature, iatrogenic interventions leading to preterm delivery were not to be included.

We acknowledge that the present meta-analysis has dealt with several limitations. Several parameters may contribute to this finding. For instance, the methodological heterogeneity that was noted in these studies may result in significant selection bias that may prohibit clear conclusions. Specifically, the two main studies that support the use of antibiotics to reduce the risk of preterm birth, namely Fuchs et al. as well as Hatanaka et al. used historical comparisons for the control group, which were recruited from populations of previous research projects. Moreover, significant imbalance among the group of controls and patients receiving antibiotics was observed denoting a possibility of attrition bias. On the other hand, Cuff et al. which recruited both groups (experimental and control) during the same study period did not note a significant association between antibiotic use and reduction of the risk of preterm birth.

Another notable limitation of the included studies is that there is no subdivision of the study groups based on the cervical length of the patients (except for Hatanaka et al.) as the screening strategy was based on the presence of amniotic fluid sludge and as such, the most important factor associated with preterm delivery could not be used for a subanalysis to be conducted. This should be a factor to be taken into consideration when future studies are being designed as it is evident by several recent studies that cervical length measurement and the gestational age at the time of admission remain the strongest predictors for preterm delivery [22].

The actual characteristics of the population that was used is worth mentioning as well. Specifically, whereas Fuchs et al. used a mixed sample of pregnant women at risk of preterm labor as well as asymptomatic women, Jin et al. used only women with present uterine contractions. Cuff et al. as well as Hatanaka et al. both used asymptomatic women with intact membranes at 15–26 weeks of gestational age. These characteristics may severely affect the probability of preterm birth and thorough examination in further studies is needed to evaluate the need for antibiotic therapy both among women with sludge in the absence of co-existing high-risk pregnancy features, as well as among those that present with risk factors for preterm birth, including reduced cervical length, laboratory features indicative of chorioamnionitis in the absence of clinical factors as well as premature contractions.

However, given the fact that first, the presence of uterine contractions could be considered a high risk factor for preterm delivery (Cuff et al.), second, that Hatanaka et al. subdivided their study groups to high risk subgroups based on the cervical length, Mullerian malformations, history of preterm birth and late miscarriages,as well as, prior cervical conization and third, that Fuchs et al. included high risk women based on the presence of threatened preterm labour and their previous obstetrical history we conducted a subanalysis for those high risk pregnancies (Table 3). It was demonstrated that in those AFS study groups, the use of antibiotics led to a statistical significant reduction of preterm delivery rates < 28 weeks,  < 32 weeks and < 34 weeks revealing that those patients might be benefited from an antibiotic focused policy.

Lastly, but equally important, it is worth mentioning that the proposed antibiotics significantly differed among studies, rendering problematic the interpretation of microbe coverage as well as the retrieval of safe data that will permit safe use in clinical studies. To be more precise Jin et al. used a combination of IV ceftriaxone (1 g/24 h), PO clarithromycin (500 mg/12 h) and IV metronidazole (500 mg/8 h) for a maximum of 4 weeks while the studies from Cuff et al. and Fuchs et al. both used PO azithromycin (500 mg/24 h) on Day 1 followed by PO azithromycin (250 mg/24 h) from Day 2 to Day 5. Lastly, Hatanaka et al. used PO clindamycin (300 mg / 6 h) and cephalexin (500 mg/6 h) for 7 days in their low-risk subgroup and a more aggressive combination of IV clindamycin (600 mg/8 h) and IV cephazolin (1gr/8 h) for 5 days followed by 5 days of PO treatment for the high-risk pregnant women. It is, thus, clear that those differences in the treatment regimens might have an influence on their reported outcomes.

### Comparison with existing literature

tion with or without concurrent infection of the membranes has been proven to be a well-documented risk factor for preterm labor and PPROM [8–10]. As such, the use of antibiotic regimens has been thoroughly investigated. Recently, Yoon et al. demonstrated that antibiotic treatment eradicated intraamniotic infection/inflammation in 79% of patients with signs of preterm labor, intact membranes and proven intraamniotic infection/inflammation by amniocentesis and led to a treatment success rate (either resolution of intraamniotic infection/inflammation or delivery > 37 weeks) of 84% in those women who remained undelivered 1 week post amniocentesis [18]. Furthermore, in order for the need for amniocentesis and its potential complications to be reduced, recent studies have synthetized a noninvasive scoring system consisting of 4 parameters (maternal serum CRP, cervical dilatation, cervical fetal fibronectin and gestational age) which appeared both sensitive and specific in predicting the underlying presence of intraamniotic infection and/or inflammation [19].

AFS has been hypothesized to be an indicator of microbial invasion in the amniotic cavity and of an ongoing inflammatory process. As denoted by our previous meta-analysis, current bibliography has associated intraamniotic infection and/or inflammation with increased risk for preterm delivery and studies have shown that antibiotic treatment can decrease the rate of preterm labor in a subset of patients with proven positive markers for intraamniotic infection/inflammation [12, 13]. As shown in the *Principal Findings* and *Strengths and Limitations* sections above, AFS has been studied as a potential risk factor for preterm delivery (in low and high risk subgroups) and chorioamnionitis with the current evidence still lacking,though, as to its clear value in different populations (high or low risk for preterm delivery) or its effect based on the gestational age of detection and further studies with bigger sample sizes are needed to address not only the issue of preterm delivery and chorioamnionitis but also the effect of antibiotics on neonatal outcomes. Neonatal ICU admission, composite neonatal morbidity and perinatal/neonatal death were assessed by Jin et al. and Cuff et al. demonstrating similar non-significant results for the former but conflicting results about the last two. Moreover, Cuff et al. and Hatanaka et al. assessed the effect of antibiotic on the weight of the newborn showing non-significant alterations in birthweights < 2500 g and < 1500 g [21].

Kusanovic et al. were the first to collect and analyse the amniotic fluid sludge itself through transabdominal amniocentesis, guiding the antibiotic treatment based on the microorganisms isolated. They concluded that it appears important to assess the amniotic fluid sludge directly given the fact that women with sludge may have negative amniotic fluid cultures even though several microorganisms may be present withing the particulate matter. [20] In their case, the patient delivered spontaneously 5 days after her admission despite the tailored antibiotic regimen. More recently, Yeo et al. demonstrated a case of complete resolution of the sonographic and speculum findings—after the administration of antibiotics—in a patient with midtrimester cervical insufficiency, sterile intra-amniotic inflammation and amniotic fluid sludge that eventually delivered after 36 weeks. In their case, the amniotic fluid demonstrated no microorganisms but IL-6 levels were high indicating a sterile intra-amniotic inflammation. By the time the results were ready (22^+3^ weeks of gestation), the cervix was 3 cm dilated and the membranes were bulging, time when the antibiotics were initiated and continued for 11 days. 10 days later (23^+6^ weeks of gestation) there was no demonstratable bulging and the cervical length was 21 mm and 3 weeks later (25^+3^ weeks of gestation) the quantity of sludge appeared smaller and the cervix was 12.7 mm long, length that was even increased later in pregnancy. The authors suggested, in accordance with the findings of Yoon et al., that in cases with second trimester cervical insufficiency with or without amniotic fluid sludge, the presence of intraamniotic infection/inflammation should be considered and antibiotics could be administered in an effort to prevent prematurity and its subsequent complications. In accordance to the above, a recent study, which assessed the risk for preterm delivery based on machine learning techniques that utilized multivariable models, showed the potential negative effect of antibiotics in prevention of prematurity in women without clinical or subclinical evidence of infection due to a likely alteration of maternal microbiome which displays a protective role [23]. It should be highlighted that this study, similar to many others recently published, represents an effort for individualization of the modern practice of medicine, aiming for a more precise and guided identification of high risk groups within the general population Table 4.

**Table 4:**
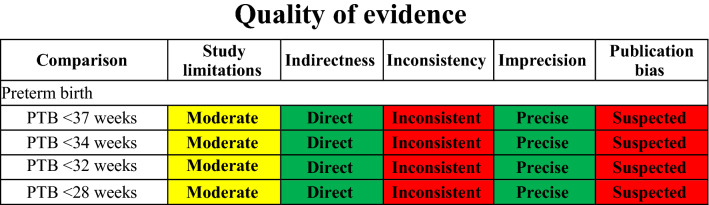
Outcomes of quality of evidence evaluation

*PTB *preterm birth

## Conclusions and implications

We acknowledge that the studies included in the present meta-analysis are quite heterogeneous in terms of methodological characteristics and baseline patient settings. Moreover, it should be noted that the number of enlisted studies/participants is rather small, therefore, leaving plenty of space for future research, as the results seem to be underpowered and not easily interpretable. Furthermore, to the moment, antibiotics seem to be helpful in a subset of patients with proven intraamniotic infection and/or inflammation, finding that necessitates a stricter stratification of the patients who will be detected with amniotic fluid sludge. Taking this information into consideration, we believe that future research should focus on the way AFS affects otherwise asymptomatic women, as well as, populations at risk of preterm birth and investigation should be carried out to detect the subsets of patients that are more likely to benefit from the sonographic detection of AFS itself,as well as, in case of detection, from its treatment. Furthermore, should research reveal that antibiotics reduce the preterm delivery rates, standardization of the proposed scheme and duration of treatment is needed to permit actual introduction in clinical practice. 

## Data Availability

The data included are available.
